# Applicability of Next Generation Sequencing Technology in Microsatellite Instability Testing

**DOI:** 10.3390/genes6010046

**Published:** 2015-02-12

**Authors:** Chun Gan, Clare Love, Victoria Beshay, Finlay Macrae, Stephen Fox, Paul Waring, Graham Taylor

**Affiliations:** 1 Department of Pathology, Faculty of Medicine, Dentistry and Health Sciences, University of Melbourne, Parkville, Victoria 3052, Australia; E-Mails: clare.love@unimelb.edu.au (C.L.); pwaring@unimelb.edu.au (P.W.); graham.taylor@unimelb.edu.au (G.T.); 2 Department of Colorectal Medicine and Genetics, Familial Cancer Clinic, Royal Melbourne Hospital, Parkville, Victoria 3050, Australia; E-Mail: finlay.macrae@mh.org.au; 3 Department of Medicine, Royal Melbourne Hospital, University of Melbourne, Parkville, Victoria 3050, Australia; 4 Department of Pathology and Sir Peter MacCallum Department of Oncology, University of Melbourne, East Melbourne, Victoria 3002, Australia; E-Mails: victoria.beshay@petermac.org (V.B.); stephen.fox@petermac.org (S.F.)

**Keywords:** microsatellite instability, next generation sequencing, colorectal cancer, MiSeq

## Abstract

Microsatellite instability (MSI) is a useful marker for risk assessment, prediction of chemotherapy responsiveness and prognosis in patients with colorectal cancer. Here, we describe a next generation sequencing approach for MSI testing using the MiSeq platform. Different from other MSI capturing strategies that are based on targeted gene capture, we utilize “deep resequencing”, where we focus the sequencing on only the microsatellite regions of interest. We sequenced a series of 44 colorectal tumours with normal controls for five MSI loci (BAT25, BAT26, BAT34c4, D18S55, D5S346) and a second series of six colorectal tumours (no control) with two mononucleotide loci (BAT25, BAT26). In the first series, we were able to determine 17 MSI-High, 1 MSI-Low and 26 microsatellite stable (MSS) tumours. In the second series, there were three MSI-High and three MSS tumours. Although there was some variation within individual markers, this NGS method produced the same overall MSI status for each tumour, as obtained with the traditional multiplex PCR-based method.

## 1. Introduction

Microsatellites are short, repetitive DNA sequences that consist of repeating mononucleotide, dinucleotide or polynucleotide sequence loci. These regions are prone to base-pair mismatches during DNA replication, but are safeguarded against these errors by the mismatch repair (MMR) proteins.

Microsatellite instability (MSI) refers to the expansion or contraction of the DNA repetitive regions. It is caused by the absence of functional MMR proteins and is a phenomenon that is observed in almost all Lynch syndrome patients [1] and up to 15%–20% of sporadic colorectal cancers [2]. Three distinct MSI phenotypes have been described. They can be subdivided into high (MSI-H) or low (MSI-L) levels of instability, with MSI-H defined as instability at ≥30%–40% of the examined loci and MSI-L defined as instability at <30% of loci [3]. Tumours are defined as microsatellite stable (MSS) if none of the examined loci demonstrate instability [3]. Clinically, MSI-L tumours behave in a manner similar to MSS tumours [3].

The mononucleotide MSI loci, BAT25 and BAT26, have the highest accuracy in predicting MSI-H tumours, with sensitivity and specificity approaching 94%–98% for both markers [4]. The quasimonomorphic [5] feature of these markers, defined as little or no polymorphism in these loci across all ethnic populations, allows the testing of tumour tissue without the need for a corresponding normal control. However, some unstable tumours may have stable BAT26 loci due to a large intragenic MSH2 deletion, causing complete absence of the BAT26 loci in the tumour tissue [6]. Other MSI loci are generally added to correctly detect these cases.

The presence of MSI-H in colorectal tumour tissues suggests MMR protein deficiency. These patients are referred for further definitive genetic testing for germline MMR mutation (Lynch syndrome) once the BRAF V600E mutation has been excluded [7]. MSI-H tumours respond poorly to 5-flurouracil-based chemotherapy, and an alternative chemotherapy regimen should be considered [8,9,10]. The demand for MSI testing is increasing, and several papers have suggested a universal approach for MSI testing in all colorectal cancers [7,11,12].

Conventional MSI testing is commonly performed using a fluorescent multiplex PCR-based method where the amplified PCR products are run on an automated capillary electrophoresis analyser, and the fragments generated are then analysed using the GeneMarker analysis software (Softgenetics LLC, State College, PA, USA). The advantage of NGS technology is that it allows massively parallel sequencing and is capable of producing millions of sequences at once [13], which usually translates to better efficiency, especially in the setting of testing large batches of colorectal tumour samples.

Here, we describe an alternative MSI testing strategy using NGS technology. Although other groups have recently developed a methodology for classifying MSI based on NGS data from targeted gene capture [14,15,16,17,18], here, we utilize “deep resequencing”, where we target the sequencing at smaller regions of interest (*i.e.*, amplicons (MSI loci)) [19]. We demonstrate an orthogonal means of identifying sequence variation by grouping the reads as amplicons prior to any alignment. The MSI amplicons sequenced are mapped against an index, generating groups of identical reads (with information on the amplicon’s nucleotide base pair length and read count). This information allows direct comparison of repeat length at the MSI loci of tumour *versus* normal tissue.

## 2. Materials and Methods

This project, designated QA2013117, was approved by the Human Research Ethics Committee, The Royal Melbourne Hospital, Grattan Street, Parkville, 3050 Victoria on 15 August 2013.

### 2.1. Study Samples

Two cohorts, Series 1 (n = 44 paired tumour and normal) and Series 2 (6 tumour samples) were recruited from Peter MacCallum Cancer Institute and Royal Melbourne Hospital, respectively. DNA extraction was performed on a total of 94 formalin fixed paraffin-embedded (FFPE) tissue samples using the Qiagen DNA extraction Kit (Qiagen, Germany). DNA quantification was performed using the Qubit dsDNA HS assay kit (Life Technologies, Victoria, Australia).

The tumours from both series were subjected to MSI testing using a fluorescent multiplex PCR-based method and target sequencing with NGS technology on the MiSeq (Illumina Inc., San Diego, CA, USA) platform. The MSI markers tested are shown in Table 1.

**Table 1:** Microsatellite instability (MSI) markers tested for both series.

Tumours	MSI markers tested	
	Multiplex PCR	NGS
Series 1 *	***BAT25 *^†^, *BAT26***, CAT25, NR21, NR22, NR24, ***D5S346***, D2S123, D17S250	***BAT25***, ***BAT26***, BAT34c4, D18S55, ***D5S346***
Series 2 **^‡^**	***BAT25*, *BAT26***, NR21, NR24, MONO27	***BAT25***, ***BAT26***

* Series 1, with normal tissue control; ^‡^ Series 2, no normal tissue control; ^†^ bold markers are included in both multiplex PCR- and NGS-based methods.

### 2.2. MSI Loci (Amplicons) Target Resequencing with MiSeq

The amplicons were generated using a 2-stage PCR approach. The first-stage PCR (inner cycle) was to isolate the microsatellite sequences of interest and was carried out according to the following conditions: 98 °C for 30 s, 15–20 cycles × (98 °C for 10 s, 58 °C 15 s and 72 °C for 20 s), 72 °C for 2 min and a final cooling step at 4 °C. The primers used are shown in Table 2 (Promega Microsatellite Instability Kit, Promega Corporation, Fitchburg, WI, USA). The primers used in the second stage PCR (outer cycle) contained sequences that bind to both the MSI sequences and the Illumina MiSeq platform. These primers were designed according to the manufacturer’s instructions (not shown) to anchor the amplicons to the MiSeq flow cell, so that they could be sequenced. They were prepared with the Nextera 96 index kit (Illumina Inc.). After the inner cycle, 2 μL of the PCR products were amplified with the outer cycle primers according to the following conditions: 98 °C for 30 s, 20 cycles × (98 °C for 10 s, 60 °C 15 s and 72 °C for 20 s), 72 °C for 2 min and a final cooling step at 4 °C. The final PCR products were purified with Ampure XP beads (New England BioLabs Inc., Ipswich, MA, USA), and their size was checked on gel (E-Gel Agarose Gel Electrophoresis System, Life Technologies, Victoria, Australia) and then loaded onto the MiSeq sequencer.

**Table 2:** Primers for the inner cycle PCR.

MSI loci	Position (chromosome)	Coordinates	Length (base pair)	Forward sequence	Reverse sequence
BAT25	4q12	55598151-55598274	123	5'-TCGCCTCCAAGAATGTAAGT-3'	5'-TCTGCATTTTAACTATGGCTC-3'
BAT26	2p	47641487-47641608	121	5'-TGACTACTTTTGACTTCAGCC-3'	5'-AACCATTCAACATTTTTAACCC-3'
BAT34c4	17p13.1	7572124-7572254	130	5'-ACCCTGGAGGATTTCATCTC-3'	5'-AACAAAGCGAGACCCAGTCT-3'
D18S55	18q22.1	61873501-61873648	147	5'-GGGAAGTCAAATGCAAATC-3'	5'-AGCTTCTGAGTAATCTTATGCTGTG-3'
D5S346	5q22.2	112213624-112213748	124	5'-ACTCACTCTAGTGATAAATCGGG-3'	5'-AGCAGATAAGACAGTATTACTAGTT-3'

### 2.3. Sequencing

The NGS runs were performed with MiSeq version 2 sequencing reagents according to the manufacturer’s recommendations.

### 2.4. Data Analysis

#### 2.4.1. Amplivar and SeqPrep for Processing NGS Data

We used an alignment-free approach to measure microsatellite length in each of the amplicons. The output from the MiSeq sequencer consists of millions of forward and reverse reads in the FASTQ format. The reads were merged using SeqPrep (https://github.com/jstjohn/SeqPrep). Merged FASTQ files were quality-filtered, where reads with a <Q30 score were discarded (a read with a ≥Q30 score indicates an error sequencing rate of <1/1,000 base pairs). They were then grouped by amplicon by matching to a lookup table corresponding to the flanking regions (MSI PCR primers) using Amplivar_MSI (Amplivar_Msi script and lookup tables, Supplementary Material). Together with the information pertaining to the read counts and microsatellite genetic sequence, these were exported to Excel spreadsheet for further analysis.

#### 2.4.2. Quantifying Amplicons

In the next analytical phase, we used Excel to further characterize the amplicons based on their read count and microsatellite length. The length of the microsatellite sequences was computed using the Excel function, LEN. With this information, we drew a column chart, with the Y-axis representing the read count and the X-axis representing the microsatellite length, and compared the expression profiling of the microsatellite loci in the tumour *versus* the corresponding normal tissue.

#### 2.4.3. Defining Individual Locus Stability

**Figure 1:**
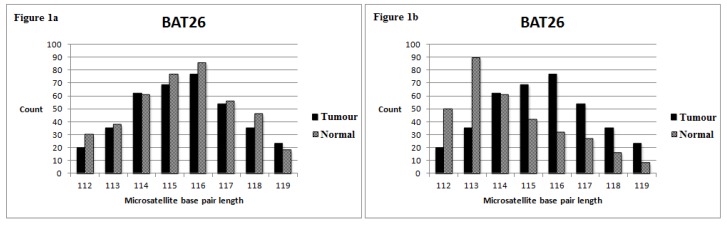
The Y-axis corresponds to the number of read counts for each amplicon; the X-axis represents the base pair length of the microsatellite region. (**a**) The tumour’s BAT26 amplicon with the highest read count has a base pair length of 116. This is similar to the corresponding normal tissue, where its BAT26 amplicon also has the most read counts at a 116-base pair length. Hence, this amplicon is deemed stable. (**b**) The tumour’s BAT26 amplicon with the highest read count has a base pair length of 113. It is 3 base pairs shorter than the BAT26 amplicon in the corresponding normal tissue. Hence, this amplicon is deemed unstable. (Definition of unstable loci: ≥2 base pair deviations for the mononucleotide marker and ≥4 base pair deviations for the dinucleotide marker).

Figure 1 demonstrates how we define unstable loci. The amplicon with the highest read count represents the microsatellite sequences that are maximally expressed in the corresponding tissue. We defined unstable loci based on a cut-off of ≥2 and ≥4 base pair deviations from the normal tissue for mononucleotide and dinucleotide MSI loci, respectively. Other cut-offs were unsuitable, as many misclassifications occurred. For example, adopting less stringent criteria (≥1 and ≥2 for mononucleotide and dinucleotide MSI loci, respectively) resulted in misclassification of 6 and 2 tumours from Series 1 and Series 2, respectively. These stable tumours were misclassified as MSI-H.

#### 2.4.4. Defining the Overall MSI Status for Each Tumour

A tumour was classified as MSI-H if 2 or more (≥40%) of the MSI loci demonstrated instability, as MSI-L if only 1 of the MSI loci demonstrated instability or as MSS if none of the MSI loci showed instability.

## 3. Results

### 3.1. MiSeq Sequencing Profile

We performed a total of four MiSeq V2 runs. The average cluster density generated on the sequencing platform ranged from 875,000 to 1,058,000 clusters/mm^2^. The average cluster passing filter per run, a value indicating the readable clusters without a signal overlap from the surrounding clusters, was 79.12%. The sequencing output per MiSeq run ranged from 1,140,000 to 1,540,000 reads with a mean of 1,400,000 reads. The average percentage of reads passing through the filter that had a sequencing quality score of >Q30 was 71.88%. The average sequencing depth ranged from 5,000 to 8,000× for each base pair per amplicon, indicating the coverage for each amplicon.

### 3.2. MSI Status of Series 1 (with Normal Tissue Control)

**Figure 2:**
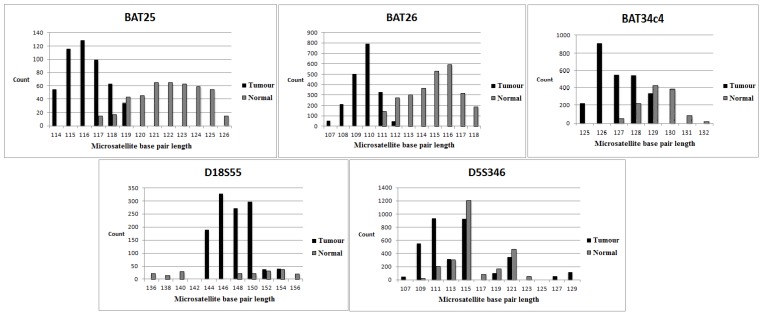
Data derived from NGS-MSI-High tumour. The upper and lower panels correspond to three mononucleotide loci and two dinucleotide loci, respectively. The tumour and normal tissue are represented by the black and grey columns, respectively. There is a deletion in the base pair length (3–6 base pairs) for each mononucleotide locus in the tumour compared to normal tissue. D18S55 demonstrates an allelic loss and deletion (8 base pairs) in the base pair length. D5S346 shows expansion and deletion (4–8 base pairs) in the base pair length.

**Figure 3:**
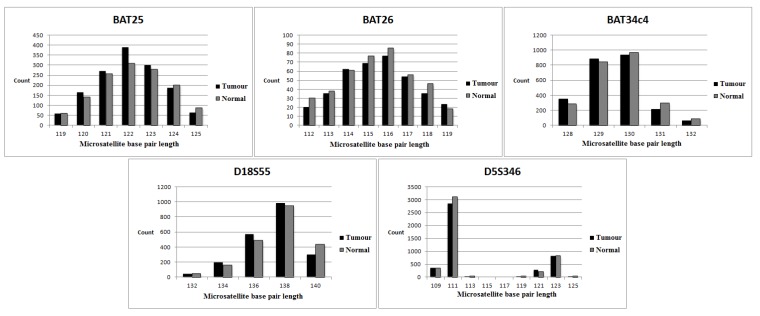
Data derived from NGS-MSI-stable tumour. The tumour and normal tissue are represented by the black and grey columns, respectively. The microsatellite base pair length for the MSI loci is similar in both the tumour and normal tissue.

Using our protocol as described in the Materials and Methods, we determined 17 MSI-H, 26 MSS and one MSI-L tumours (Table 3). The overall MSI results for all tumours were 100% concordant with the multiplex PCR-based method (data not shown [20,21]) only when we grouped MSS and MSI-L tumours together. In Case 21, the D18S55 locus was unstable by NGS (MSI loci for the multiplex PCR-based method were all stable; D18S55 was not included in the multiplex PCR panel), resulting in a classification of MSI-L rather than MSS. Examples of MSI-H and MSS tumours are shown in Figure 2 and Figure 3.

### 3.3. MSI Status of Series 2 (No Normal Tissue Control)

Three MSI-H tumours demonstrated instability in both BAT25 and BAT26 mononucleotide MSI loci (Table 4). In Case 5, both the markers were stable by NGS, resulting in a classification of MSS rather than MSI-L (the MSI loci for the multiplex PCR-based method were all stable, except NR21, resulting in the classification of MSI-L; NR21 was not included in the NGS panel). The remaining MSS tumours were stable in these loci.

### 3.4. Evaluation of Individual Markers (NGS *versus* the Multiplex PCR-Based Method)

We compared the MSI loci that were derived from NGS with the gold standard, the traditional multiplex PCR-based method. The result of any individual MSI locus from NGS was treated as incorrect if it did not match the multiplex PCR-based method. A comparison could not be made for BAT34c4 and D18S55 for Series 1, as the Peter MacCallum laboratory did not test these loci. The NGS results of BAT25 and BAT26 were 100% accurate when compared to traditional MSI testing, yielding 100% sensitivity (95% CI 83.2–100) and 100% specificity (95% CI 88.4–100). The dinucleotide loci, D5S346, showed 66.7% sensitivity (95% CI 29.9–92.5) and 94.3% specificity (95% CI 80.8–99.3). There were three false negative results out of nine unstable loci and two false positive results out of 35 stable loci (Table 3). These results did not affect the overall MSI status for each tumour sample. The MSI loci in Series 2 showed 100% accuracy in terms of sensitivity (95% CI 29.2–100) and specificity (95% CI 29.2–100).

**Table 3:** MSI status of individual loci for Series 1 (with normal tissue control). An unstable locus is defined by a cut-off of ≥2 and ≥4 base pair deviations from the normal tissue for mononucleotide and dinucleotide markers, respectively. MSI phenotypes: MSI-stable, none of the loci demonstrating instability; MSI-Low, MSI at only one locus; MSI-High, MSI at two or more loci. A comparison of individual markers (NGS *versus* multiplex PCR) was only made with BAT25, BAT26 and D5S346. Abbreviations: M, mononucleotide; D, dinucleotide; FN, false negative; FP, false positive; +, unstable locus; −, stable locus.

Cases	BAT25 (M) (NGS/ Multiplex PCR)	BAT26 (M) (NGS/ Multiplex PCR)	D5S346 (D) (NGS/ Multiplex PCR)	BAT34c4 (M) (NGS only)	D18S55 (D) (NGS only)	MSI status (NGS)	MSI status (Multiplex PCR)
1	−/−	−/−	−/−	−	−	Stable	Stable
2	−/−	−/−	−/−	−	−	Stable	Stable
3	+/+	+/+	+/+	+	−	High	High
4	+/+	+/+	+/+	+	+	High	High
5	−/−	−/−	−/−	−	−	Stable	Stable
6	+/+	+/+	−/−	−	+	High	High
7	−/−	−/−	−/−	−	−	Stable	Stable
8	+/+	+/+	+/+	+	+	High	High
9	+/+	+/+	−/−	−	−	High	High
10	+/+	+/+	−/−	+	+	High	High
11	−/−	−/−	−/−	−	−	Stable	Stable
12	−/−	−/−	−/−	−	−	Stable	Stable
13	−/−	−/−	−/−	−	−	Stable	Stable
14	−/−	−/−	−/−	−	−	Stable	Stable
15	+/+	+/+	−/−	+	+	High	High
16	−/−	−/−	−/−	−	−	Stable	Stable
17	−/−	−/−	−/−	−	−	Stable	Stable
18	+/+	+/+	−/+ FN	+	−	High	High
19	+/+	+/+	+/+	+	+	High	High
20	−/−	−/−	−/−	−	−	Stable	Stable
21	−/−	−/−	−/−	−	+	Low	Stable
22	−/−	−/−	−/−	−	−	Stable	Stable
23	−/−	−/−	−/−	−	−	Stable	Stable
24	−/−	−/−	−/−	−	−	Stable	Stable
25	−/−	−/−	−/−	−	−	Stable	Stable
26	+/+	+/+	+/+	−	+	High	High
27	+/+	+/+	−/−	+	+	High	High
28	−/−	−/−	−/−	−	−	Stable	Stable
29	+/+	+/+	−/−	+	−	High	High
30	−/−	−/−	−/−	−	−	Stable	Stable
31	−/−	−/−	−/−	−	−	Stable	Stable
32	+/+	+/+	−/+ FN	−	−	High	High
33	−/−	−/−	−/−	−	−	Stable	Stable
34	+/+	+/+	−/+ FN	+	−	High	High
35	−/−	−/−	−/−	−	−	Stable	Stable
36	−/−	−/−	−/−	−	−	Stable	Stable
37	+/+	+/+	+/− FP	+	+	High	High
38	−/−	−/−	−/−	−	−	Stable	Stable
39	−/−	−/−	−/−	−	−	Stable	Stable
40	−/−	−/−	−/−	−	−	Stable	Stable
41	−/−	−/−	−/−	−	−	Stable	Stable
42	+/+	+/+	+/+	+	−	High	High
43	+/+	+/+	+/− FP	+	+	High	High
44	−/−	−/−	−/−	−	−	Stable	Stable

**Table 4:** MSI status of individual loci for Series 2 (no normal tissue control). An unstable locus is defined by a cut-off of ≥2 base pair deviations from the normal tissue for mononucleotide markers. All MSI-H (high metastasis) tumours demonstrated instability in both BAT25 and BAT26 MSI loci. Abbreviations: M, mononucleotide; +, unstable locus; −, stable locus.

Cases	BAT25 (M) (NGS/Multiplex PCR)	BAT26 (M) (NGS/Multiplex PCR )	MSI status (NGS)	MSI status (Multiplex PCR)
1	+/+	+/+	High	High
2	+/+	+/+	High	High
3	−/−	−/−	Stable	Stable
4	+/+	+/+	High	High
5	−/−	−/−	Stable	Low
6	−/−	−/−	Stable	Stable

### 3.5. Sensitivity and Specificity of Individual MSI Loci According to Overall MSI Status (Figure 4)

Both mononucleotide BAT25 and BAT26 MSI loci were unstable in all MSI-H tumours and were stable in all MSI-L or MSS tumours, yielding 100% sensitivity (95% CI 83.2–100) and 100% specificity (95% CI 88.4–100). BAT34c4 has a sensitivity of 76.5% (95% CI 50.1–93.2) and a specificity of 100% (95% CI 87.2–100). Both dinucleotide markers performed the worst, with 58.8% (95% CI 32.9–81.6) and 47.1% (95% CI 23–72.2) sensitivity for both D18S55 and D5S346, respectively. However, D18S55 and D5S346 achieved a specificity of 96.3% (95% CI 81–99.9) and 100% (95% CI 87.2–100), respectively.

**Figure 4:**
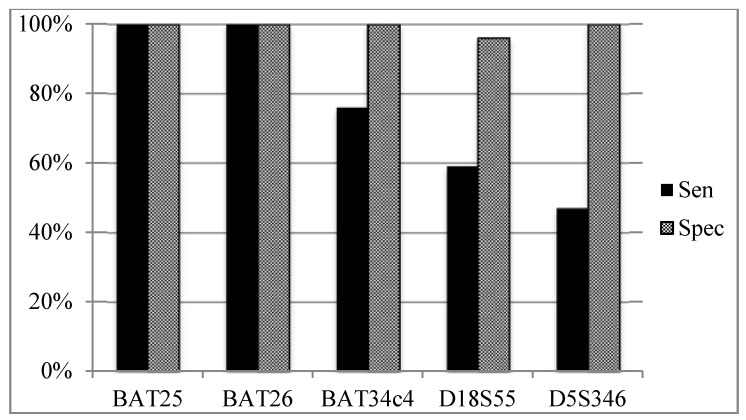
Comparison of the performance of individual loci of all cases. Abbreviations: Sen, sensitivity; Spec, specificity.

## 4. Discussion

Moving into an era of personalised genomic medicine, there is a need for technology that is reliable and efficient for gene sequencing. To date, there have been many genetic testing platforms that were developed based on NGS technology. This was related to NGS’s capability of massively parallel sequencing, which usually translates to improved efficiency in genetic sequencing compared to other traditional genetic testing platforms. Rajyalakshmi *et al.* have applied NGS technology on routine molecular testing for acute myeloid Leukaemia in the management of haematological patients [22]. There have been similar works done for inherited cardiac arrhythmias and a variety of solid tumours [23,24].

We demonstrated the applicability of NGS technology in MSI testing. Our results were 100% accurate when compared to the traditional method for MSI testing in terms of overall MSI status, where we were able to detect all MSI-H tumours using the five-panel MSI loci. As with previous studies [5,25,26], we found that instability in BAT25 and BAT26 MSI loci was highly predictive of MSI-H tumours, that 100% of our MSI-H tumours showing instability in both loci. Nevertheless, other MSI loci were included for testing, as BAT26 loci could undergo a large deletion in MSI-H tumour [6]. The dinucleotide MSI loci in our assay have a sensitivity ranging from 47%–59%, a result consistent with other studies, demonstrating that the dinucleotide markers performed less well than the mononucleotide markers [4,27].

The utility of NGS in MSI testing has been tested by several groups previously [14,15,16,17,18], where their principal method of MSI capturing strategies was based on targeted gene capture sequencing. Our MSI capturing strategy was different from these studies: instead, we utilized a methodology based on ultra-deep sequencing by focusing our capture design on the MSI loci of interest that would provide sufficient information to infer MSI status. Furthermore, other targeted gene capture sequencing (e.g., whole exome sequencing) approaches generally require NGS machinery with significantly higher throughput capability, and MSI testing is unlikely to be the sole indication to utilize such expensive strategies, especially in the context of population MSI screening, to guide subsequent germline testing for mismatch repair gene mutation. In contrast, our sequencing method utilised the smaller NGS machinery, MiSeq, which is less costly than other high throughput platforms and may be more suitable in this context. Although our strategy was based on five markers, it is flexible and can be readily expanded to include more MSI loci by designing primers that would isolate the region of interest, as described above in Section 2.2, an important feature that would allow laboratories to customise their own MSI panel. Our method lifts existing, well-characterised MSI markers and PCR amplicons into an NGS framework and leverages the cost efficiency of high throughput sequencing to deliver a tool with equivalent or superior performance to capillary analysis with a direct digital output of data in a format that is amenable to large-scale studies. Furthermore, we obtain a direct clonal readout of each microsatellite’s repeat size and sequence at the single molecule level, not an aggregate of size distribution, as delivered by the capillary method.

Data analysis remains a great challenge in NGS. Our approach was different from the conventional whole genome or exome analysis, where we developed a streamlined data processing pipeline that was easy to operate and did not require large computational infrastructure. We simplified the analysis by arranging the millions of sequences into groups by matching to a lookup table corresponding to the flanking regions (MSI PCR primers). In this circumstance, genome-wide alignment of each read was not required, hence reducing the computational burden, where we can easily perform the analysis with standard desktop machines based on the quantitative approach as described above. In contrast, other studies relied on targeted gene capture that used genomic alignment to process the NGS data [23,24,25,26,27], which may require more complex analysis and effort to derive the MSI status.

Our adopted definition to infer instability in an individual MSI locus was consistent amongst all of the samples tested. The less stringent criteria (≥1 and ≥2 for mononucleotide and dinucleotide markers) resulted in eight false positive results from Series 1 and 2, implying the difficulties of accurate sequencing in these repeated nucleotide regions. This observation was supported by the parameters derived from the MiSeq, where a significant proportion of the reads (an average of 28% of the total reads) was filtered and did not pass the Q30 score. This problem persisted despite using Q5 Hot Start High Fidelity DNA Polymerase (New England Biolabs Inc.). The sequencing errors were consistent in each of the four MiSeq runs, where the Q30 score for each read started to decline after 100 to 150 cycles. Consequently, higher coverage per MSI loci per sample was needed in anticipation of significant read loss, limiting the amount of samples that can be loaded onto each MiSeq run. The inconsistency of D5S346 loci observed between NGS and multiplex PCR-based MSI testing could also be related to this issue, where more errors were likely to occur in dinucleotide repeating regions than mononucleotide regions. This finding should be confirmed with larger studies. Nevertheless, we were able to correctly identify all of the MSI-H tumours using a higher cut-off (>2 and >4 for mononucleotide and dinucleotide markers), but this definition is subject to change, depending on the MSI loci that will be included in the capture design. Our experience showed that we achieved adequate coverage for five MSI loci up to 15 paired tumour and normal samples per run.

The average turnaround time per batch of samples (up to 15 samples per run), including pre-NGS preparation, genomic sequencing and data analysis, was approximately 24 to 32 hours. To break this down, the pre-NGS preparation (including two-stage PCR, purification of PCR products, concentration normalization and loading the PCR products onto MiSeq) took about six hours, and the data analysis took 15 to 20 minutes per sample. The majority of the time (12 hours to 16 hours) was spent on sequencing with MiSeq, which is a totally automated process. Furthermore, our assay only required 5–20 ng of extracted FFPE DNA per sample. These attributes make our assay highly desirable, especially in laboratory processing of large amounts of MSI testing.

Our study is not without limitations. Although the results were reproducible, part of the analysis was manually performed in Excel and may not be ideal from a workflow perspective. We were not able to multiplex all of the MSI locus-specific primers together despite repeated efforts to adjust the primers’ concentration and PCR conditions. Further work should focus on developing a PCR multiplex incorporating all of the MSI-locus primers of interest. In addition, in this proof of principle article, we have not performed a cost-benefit analysis to evaluate various MSI testing strategies, and this warrants further work.

In summary, our findings suggested that NGS technology is applicable in the context of MSI testing. In the face of increasing demand for MSI testing, the massively parallel sequencing ability of NGS coupled with streamlined data processing using the Amplivar and SeqPrep programs gives us an opportunity to test large batches of colorectal samples efficiently.

## 5. Conclusions

MSI has become increasingly important in a variety of clinical applications, including screening for Lynch syndrome and providing a guide for clinicians to prognosticate colorectal cancer and predict a tumour’s chemo-responsiveness to a 5-fluorouracil-based regimen [8,9,10]. Based on the “deep re-sequencing” approach, we proved that NGS is a suitable testing platform for MSI testing, where we were able to identify all of the unstable tumours using a panel of five MSI loci (BAT25, BAT26, BAT34c4, D18S55, D5S346). Our approach is unique and is different from other studies, which have mainly described a targeted gene capturing strategy for MSI testing. The latter strategies are generally more expensive, as higher throughput sequencing machines and complex data processing pipelines are required. Combining the quantitative approach, an automated streamlined data processing pipeline that is genome alignment-free and an MSI panel that is customizable, we believe our strategy is a promising alternative to the conventional multiplex PCR-based method.

## Supplementary Files

Supplementary File 1
